# Geographical resistome profiling in the honeybee microbiome reveals resistance gene transfer conferred by mobilizable plasmids

**DOI:** 10.1186/s40168-022-01268-1

**Published:** 2022-05-03

**Authors:** Huihui Sun, Xiaohuan Mu, Kexun Zhang, Haoyu Lang, Qinzhi Su, Xingan Li, Xin Zhou, Xue Zhang, Hao Zheng

**Affiliations:** 1 College of Food Science and Nutritional Engineering, China Agricultural University, Beijing, 100083 China; 2 Key Laboratory for Bee Genetics and Breeding, Jilin Provincial Institute of Apicultural Sciences, Jilin, 132000 China; 3 Department of Entomology, College of Plant Protection, China Agricultural University, Beijing, 100083 China

**Keywords:** *Apis cerana*, *A. mellifera*, Gut resistome, Antibiotic resistance genes, Horizontal transfer, IncQ

## Abstract

**Background:**

The spread of antibiotic resistance genes (ARGs) has been of global concern as one of the greatest environmental threats. The gut microbiome of animals has been found to be a large reservoir of ARGs, which is also an indicator of the environmental antibiotic spectrum. The conserved microbiota makes the honeybee a tractable and confined ecosystem for studying the maintenance and transfer of ARGs across gut bacteria. Although it has been found that honeybee gut bacteria harbor diverse sets of ARGs, the influences of environmental variables and the mechanism driving their distribution remain unclear.

**Results:**

We characterized the gut resistome of two closely related honeybee species, *Apis cerana* and *Apis mellifera*, domesticated in 14 geographic locations across China. The composition of the ARGs was more associated with host species rather than with geographical distribution, and *A. mellifera* had a higher content of ARGs in the gut. There was a moderate geographic pattern of resistome distribution, and several core ARG groups were found to be prevalent among *A. cerana* samples. These shared genes were mainly carried by the honeybee-specific gut members *Gilliamella* and *Snodgrassella*. Transferrable ARGs were frequently detected in honeybee guts, and the load was much higher in *A. mellifera* samples. Genomic loci of the bee gut symbionts containing a streptomycin resistance gene cluster were nearly identical to those of the broad-host-range IncQ plasmid, a proficient DNA delivery system in the environment. By in vitro conjugation experiments, we confirmed that the mobilizable plasmids could be transferred between honeybee gut symbionts by conjugation. Moreover, “satellite plasmids” with fragmented genes were identified in the integrated regions of different symbionts from multiple areas.

**Conclusions:**

Our study illustrates that the gut microbiota of different honeybee hosts varied in their antibiotic resistance structure, highlighting the role of the bee microbiome as a potential bioindicator and disseminator of antibiotic resistance. The difference in domestication history is highly influential in the structuring of the bee gut resistome. Notably, the evolution of plasmid-mediated antibiotic resistance is likely to promote the probability of its persistence and dissemination.

Video Abstract

**Supplementary Information:**

The online version contains supplementary material available at 10.1186/s40168-022-01268-1.

## Background

The overuse of antibiotics has led to environmental contamination through landfills, treated wastewater draining, and waste from livestock farms. The widespread use of antibiotics imposes a selection force for disseminating antibiotic resistance genes (ARGs) among bacteria [1]. ARGs carried by environmental microorganisms are picked up by animals and are potentially transferred into their native associated bacteria [2]. Recent surveillance revealed a transmission of NDM-beta-lactamase-producing bacteria from a poultry farm to wild birds [3]. *Escherichia coli* producing extended-spectrum β-lactamase have been detected from the gut of wild gulls feeding on human waste ashore [4]. Clinically relevant resistance genes were also reported in migratory birds with low human contact and transmitted geographically distantly [5]. Thus, animals not only mirror the presence of ARGs in the contaminated environment but also serve as possible reservoirs and potential vectors of multidrug-resistant microbes [6].

Wildlife and insects have been investigated as potential indicators of environmental dissemination of AMRs [7]. Specifically, honeybees are important plant pollinators, playing a fundamental role in the pollination of plant species in both natural ecosystems and agricultural crops. During pollinating within an area of ~ 3 km from the hive, honeybees interact with environmental microorganisms. The environmental antibiotic-resistant bacteria and the associated ARGs may transfer between the environmental and gut bacteria [8]. Such transfer potentially contribute to the spread of ARGs to new pollination sites during foraging trips [9]. Tetracyclines have been used in the control of American foulbrood in *A. mellifera*, which is caused by the pathogen *Paenibacillus larvae*. These treatments have led to severe accumulation of tetracycline resistance genes in the bee gut microbiome [10]. Moreover, the *tetR* genes are consistently associated with mobile elements showing high similarity to those characterized by human pathogens or domesticated animals, indicating an intermediate role of honeybees in the environmental transmission of ARGs [11]. In addition, the sulfonamide resistance gene *sul2* was detected in the bee gut, which had the highest sequence similarity to the IncQ plasmid [12]. IncQ is a family of plasmids with a unique strand-displacement mechanism of replication and functions within a broad range of hosts [13]. These results suggest that IncQ plasmids might mediate ARG transfer in the bee gut.

The honeybee *Apis cerana* represents an important pollinator in Asia; however, its gut resistome remains uncharacterized, in contrast to multiple studies on *A. mellifera*. Both *A. cerana* and *A. mellifera* have simple and host-restricted gut bacteria dominated by only 5–9 core bacterial genera. The core gut members, including *Gilliamella*, *Snodgrassella*, *Bartonella*, *Bifidobacterium*, and *Lactobacillus* Firm-4 and Firm-5, account for > 95% of the whole gut community [14]. However, bacteria of the same phylotype were observed to cluster separately, corresponding to the host species, leading to varied gut microbial compositions between *A. cerana* and *A. mellifera* [15]. It has been shown that the gut bacteria from the same genus isolated from honeybees and bumblebees differed in their ARG carriage profile [16]. Specifically, bacterial strains isolated from Chinese bumblebees possessed the multidrug resistance gene *emrB*, while tetracycline resistance genes were uniquely present in gut bacteria from the USA. Unlike *A. mellifera*, *A. cerana* were mostly domestically managed without constant transfer for pollination in China, and the traditional beekeeping practices of *A. cerana* maintain a semiferal nature less affected by artificial domestication [17]. Thus, the gut of *A. cerana* represents a promising model to evaluate the local ARG burden driven by long-term selective pressures [18]. Moreover, the conserved microbiota makes the honeybee a tractable, realistic, and confined ecosystem for studying the transfer and maintenance of ARGs across gut bacteria [19].

Therefore, honeybee may serve as an ideal indicator of the environmental antibiotic resistance. Here, we examined whether honeybees from different geographical locations exhibited distinct resistome profiles, and the potential horizontal transfer of ARGs among honeybee gut symbionts. Bee samples of *A. cerana* were collected from 14 geographical locations across China. Using metagenomic sequencing, we showed that the *A. cerana* gut resistome was dominated by different types of ARGs from those in *A. mellifera*, and the load and diversity varied for samples from different locations. The bee gut symbiotic members carried several core ARGs prevalent in samples from all locations. Transferrable ARGs were frequently detected, and streptomycin resistance loci were identified in the genomic regions of different symbionts from multiple areas. Finally, we confirmed the conjugative transfer of ARGs mediated by the mobilizable plasmid among the core bacterial genera *Gilliamella*, *Snodgrassella*, and *Bartonella*, which were specific to the honeybee gut environment. These results clarify the horizontal gene transfer among gut symbionts in the spread of antibiotic resistance.

## Methods

### Sample collection and DNA extraction

**Fig. 1:**
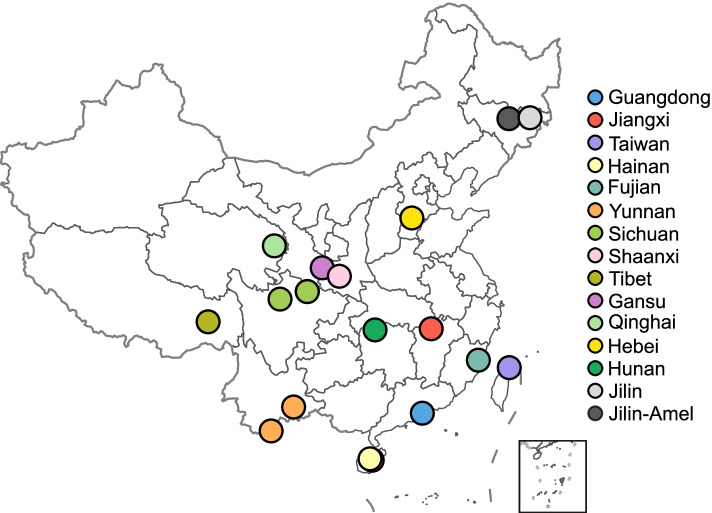
The map showing the location of 18 sampling sites from 14 provinces across China. Detailed information of the geographic location and sampling time is shown in Dataset S1

*A. cerana* bees were obtained from 18 different apiaries in 14 provinces across China (Fig. 1). We sampled worker bees from each colonies between April 2017 and January 2019. *A. mellifera* bees were collected in Jilin in September 2017. All hives of *A. cerana* and *A. mellifera* were from traditionally managed stationary apiaries, where beekeepers do not exchange the queens and domesticate without transfer. There was not any antibiotic treatment history in all hives sampled in this study. We sampled 5–10 honeybees from each apiary for the metagenomic analysis. A full list with detailed information was summarized in Dataset S1. The entire gut was pulled out from the tail of adult bees without touching the abdomen surface using sterilized forceps [20]. Individual guts were stored in 1.5-ml tubes with 100% ethanol or directly frozen at – 80 °C and were transported to the laboratory.

### DNA extraction and shotgun metagenomic sequencing

The whole gut DNA was extracted using the CTAB method described by Kwong et al. [21]. A total of 126 honeybee gut samples were used for metagenomic sequencing. Sequencing libraries were generated using NEBNext UltraTM II DNA Library Prep Kit for Illumina (New England Biolabs, MA, USA), and the library quality was assessed on Qubit 3.0 Fluorometer (Life Technologies, Grand Island, NY) and Agilent 4200 (Agilent, Santa Clara, CA) system. The libraries were then sequenced on the Illumina Hiseq X-Ten platform with 150 bp paired-end reads. Metagenomic sequencing generated 739 Gb of data with an average of 5.86 Gb for each sample. Adaptor trimming and quality control of the raw sequencing data were carried out using the fastp software (-q 20, -u 10) [22]. The reads were then mapped to the reference genomes of *A. cerana* (GCA_001442555) and *A. mellifera* (GCA_003254395) accordingly using the BWA-MEM algorithm with the option “-t 4, -R, -M” [23] to filter out sequencing reads.

### Microbiome and resistance gene analysis

The bacterial community profiling was performed using the Metagenomic Intra-Species Diversity Analysis System (MIDAS) pipeline as described in Su et al. [24]. The Shannon and Simpson indices were calculated by the ‘vegan’ package. The principal coordinates analysis (PCoA) was performed based on Bray-Curtis distance using the ‘vegan’ package. We characterized the resistome structure by aligning reads to the MEGARes database following the AmrPlusPlus pipeline [25] with modifications. Sequencing reads were mapped to the MEGARes database using the BWA algorithm (-t 10, -R). The SAM formatted alignment file was analyzed using ResistomeAnalyzer (80% nucleic acid sequence similarity) for the quantification of ARGs. The abundance of different resistance types was calculated at different levels (Gene, Group, Mechanism, and Class) corresponding to the annotation in the MEGARes database. The sequencing read abundance of ARGs was then normalized by the number of reads mapped to the 16S rRNA genes. Thus, the normalized abundance of ARGs was transformed as a “copy of ARG per copy of 16S rRNA gene” as suggested by Li et al. [26]. The number of the total 16S rRNA gene sequences was determined by METAXA2 (-f q, -plus T) [27]. To specifically detect transferrable ARGs in our metagenomic data, we analyzed our assembled contigs with ResFinder [28]. A threshold of at least 90% similarity and 60% of the reference length was used.

The bacterial origin of ARGs was predicted by assigning taxonomy to metagenomic-assembled contigs harboring antibiotic resistance genes. We assembled clean reads using MEGAHIT (-r, -t) to obtain contigs. To exclude the contigs without ARGs, we aligned the reads previously mapped to the ARGs in the MEGARes database (see above) to the assembled contigs with BLASTn, and only contigs possessing ARGs were applied for the taxonomy classification. The taxonomy of the assembled contigs containing ARGs was determined by comparing to the custom genomic database of both honey and bumble bee gut bacteria [29] using the two-way reciprocal best hit analysis.

We used Procrustes analysis to determine the correlation of resistome profiles and the microbiota compositions of each sample as described by Munk et al. [30]. We performed Hellinger transformation of the matrices of the gene-level ARG composition and the species-level microbiota taxonomy and calculated the Bray-Curtis dissimilarities between each pair of data (ARGs-Microbiota composition). The symmetric Procrustes correlation coefficients between the dissimilarity matrix of microbiota and resistome ordinated by PCoA were analyzed using the ‘protest’ function in the vegan R package.

### Antibiotic susceptibility testing and conjugation experiment

Strains of the honeybee gut bacteria, *Gilliamella apicola*, *Gilliamella apis*, *Bartonella apis*, and *Snodgrassella alvi*, were isolated from the gut of *A. mellifera* [29]. The core gut bacteria of bee guts were obtained by plating the gut homogenates on Brain Heart Infusion (BHI; Oxoid, Basingstoke, UK)) or Columbia agar medium supplemented with 5% (vol/vol) defibrinated sheep blood (Solarbio, Beijing, China) at 35 °C under a CO_2_-enriched atmosphere (5%) for 2 days. The identification of the isolated single colonies was performed by sequencing the 16S rRNA genes. The occurrence of type IV secretion system (T4SS)-associated genes necessary for the conjugation of the IncQ plasmid was detected by a BLASTn search using SecReT4 v2.0 [31]. *E. coli* MFD*pir* strain was cultivated on LB agar supplemented with 0.3 mM diaminopimelic acid at 37 °C for 24 h. We determined the antibiotic susceptibility of the bee gut bacterial strains towards different antibiotics, including chloramphenicol, kanamycin, ampicillin, and spectinomycin. Bacterial cells were grown and diluted to 10^7^ CFU/ml. We inoculated 5 μl of the bacterial culture on plates supplemented with a gradient concentration of each antibiotic. Each bacterial strain was tested in triplicates, and plates without antibiotics were used as control. The inhibition of antibiotics on different strains was determined by incubating plates for 48 h. The details of strain resistance to antibiotics are shown in Dataset S5.

The in vitro conjugation assays used either *E. coli* MFD*pir* or *G. apis* W8126 as the donor strains and other honeybee gut bacteria (*Gilliamella apis*, *Gilliamella apicola*, *Snodgrassella alvi*, *Bartonella apis*) as the recipient strains. To generate donor strains, we transferred the IncQ plasmid pBTK519 (Addgene Plasmid #110603) with the kanamycin resistance gene into *E. coli* MFD*pir* and *G. apis* W8126 via conjugation [32]. The donor strains were grown on LB or BHI agar plates with 50 μg/ml kanamycin. Cells were harvested from the plates, suspended in 1 ml of 1 × PBS, and then centrifuged for 5 min at 6000×*g*. The supernatants were removed, and the cells were washed by 1 × PBS twice. Washed cells were resuspended in PBS to a final concentration of 10^8^ CFU/ml. The donor and recipient strains were mixed in a 1:1 ratio for the conjugation experiment. Thirty microliters of the mixtures were spotted onto BHI agar containing 0.3 mM diaminopimelic acid (DAP) and incubated for 16 h. After incubation, we scraped the cell mixture into a 1.5-ml centrifuge tube and washed twice with 1 ml sterile 1 × PBS to remove residual DAP. Then, we plated 100 μl of the mixtures on selective plates with 25 μg/ml of kanamycin and the other designated antibiotics for different recipient strains (Dataset S5). Candidate transconjugants were picked and passaged again on selective medium. The identity was confirmed by PCR amplification and Sanger sequencing of the 16S rRNA gene. All the conjugative mating experiments were conducted in three biological triplicates.

## Results

### Regional distribution of the *A. cerana* gut resistome across China

A total of 94 gut samples of *A. cerana* were collected from 14 provinces across China (5–10 guts from each sampling site). We collected samples from two different sites in Hainan, Yunnan, and Sichuan provinces (Fig. 1; Dataset S1). To compare the resistome patterns of *A. cerana* and *A. mellifera*, we also collected 32 guts of *A. mellifera* from Jilin Province. These *A. mellifera* colonies were domesticated without transportation for pollination. After filtering reads derived from bee hosts, we obtained an average of 12 million reads (150 bp paired-end) per sample by shotgun metagenomic sequencing. First, we analyzed the gut community structure of both bee species sampled in this study. Overall, the gut microbiota of both *A. mellifera* and *A. cerana* was dominated by a few core bacterial genera, as described in previous studies [21]. The genus *Apibacter* specific to eastern honeybees and bumblebees was detected only in the *A. cerana* gut, while *Bartonella* was more abundant in *A. mellifera* (Figure S1a). The diversity of the gut community of *A. mellifera* from Jilin was higher than that of *A. cerana* sampled from the same area and most *A. cerana* from other locations (Figure S1b, c). PCoA showed that the microbiomes of *A. mellifera* formed a separate cluster with those of *A. cerana* (Figure S1d). Detailed analyses of the gut composition of *A. cerana* have been included in a separate study [24].

**Fig. 2:**
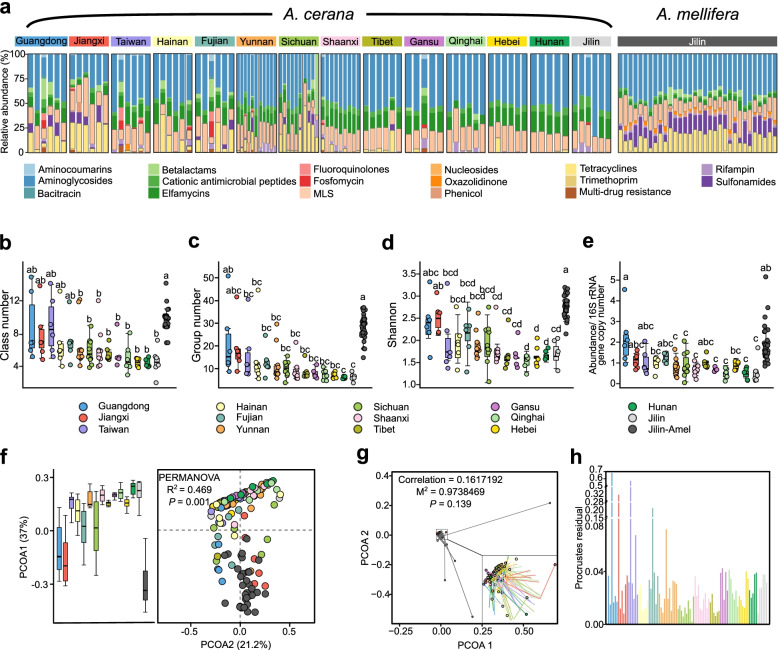
Abundance and composition of ARGs in *A. cerana* and *A. mellifera* guts. **a** Stacked bar charts showing relative abundance of different classes of ARGs in each bee individual. **b**, **c** Box plots showing the total class (**b**) and group **c** number of ARGs identified in each sample. **d** The total abundance of ARGs normalized by the amount of gut bacterial cells. **e** The Shannon diversity of each sample at the level of ARG group. **f** PCoA based on Bray-Curtis distances of group-level resistome compositions. Boxplots (left panel) indicate the distribution of each group of honeybee along the first principal coordinate (PCoA 1). **g**, **h** Procrustes analysis between the microbiota taxonomic compositions and the profiles of resistome. **g** The change in the ordination position of resistome (dotted ends) and the microbiota (non-dotted ends) is displayed. The correlation coefficients and significance were calculated with the protest function in vegan. *M*^**2**^ indicates the sum of squared distances between matched sample pairs. **h** The residual line plot enables easier residual size comparison, showing the differences in the microbiome-resistome association between *A. cerana* gut samples. Different letters (a, b, c, d) above each bar stand for statistical differences between sampling sites (least-significant difference (LSD) test, *P* < 0.05)

We used the AMR++ pipeline to analyze the ARG composition of each sample. Overall, the guts of *A. cerana* and *A. mellifera* harbored 78 and 306 groups of ARGs, respectively, presumably conferring resistance to 37 and 26 classes of antibiotics (Dataset S2). ARGs conferring resistance to classes of aminoglycosides, elfamycins, macrolide-lincosamide-streptogramin (MLS), and cationic antimicrobial peptides were present in almost all bees (Fig. 2a)*.* The ARGs of aminoglycosides and MLS were dominant in the majority of *A. cerana* samples, while *A. mellifera* from Jilin had a larger proportion of ARGs belonging to the tetracycline (16.8%) and sulfonamide (13.2%) families. Although ARGs of tetracyclines were not abundant in most *A. cerana* guts, they were prevalent in samples from Guangdong, Jiangxi, Hainan, Fujian, and Sichuan provinces (Figure S2). The abundance of ARGs of aminocoumarins, bacitracin, fluoroquinolones, fosfomycin, nucleosides, trimethoprim, and multidrug resistance was low in both bee species, which all showed an average abundance < 7%.

The diversity of ARGs at the levels of classes and groups (defined by the AMR++ database) [25] was not significantly different between most *A. cerana* samples, but they were higher in *A. mellifera* than in *A. cerana* from several locations (Fig. 2b, c). Specifically, the average group number of ARGs in *A. mellifera* was more than 4 times higher than that in *A. cerana*, and both of these bees were collected from Jilin (Fig. 2c). Consistently, *A. cerana* samples showed lower Shannon diversity indices than *A. mellifera*, except in Guangdong and Jiangxi (Fig. 2d). To exclude the impact of the size of gut bacterial numbers, the relative abundance of ARGs was then normalized by the copy number of 16S rRNA genes. The ARG load was significantly higher in the gut of *A. mellifera* than in that of *A. cerana* collected from Jilin and those from many other locations (Fig. 2d). PCoA based on the Bray-Curtis distance showed that when plotted by sampling sites and host species, the resistome of all sampled honeybees exhibited clear segregation (Fig. 2f). Notably, the gut resistome composition of *A. mellifera* was isolated from the *A. cerana* samples (Fig. 2f, left panel). Since the resistome patterns might be influenced by the composition and diversity of gut microbiota, we wondered if the varied resistome of honeybees was primarily affected by the microbiota structure. Procrustes analysis indicated that samples with similar taxonomic compositions did not necessarily show similar resistome patterns (*P* = 0.139, Fig. 2g), and the Procrustes residuals were extremely high in samples from Guangdong, Jiangxi, Taiwan, and Fujian provinces (Fig. 2h), indicating that the resistome was not determined merely by the bacterial composition in *A. cerana*.

Since regional factors cannot be excluded as the cause of the differences in the gut resistome of the two honeybee species, we further assessed the distribution of ARGs in the public metagenomic datasets of *A. cerana* and *A. mellifera* sampled from Japan and Switzerland [33]. The resistomes of both honeybee species from all locations were dominated by aminoglycoside resistance genes (Figure S2). All *A. mellifera* samples had a higher ratio of tetracycline resistance genes than the *A. cerana* samples. The tetracycline resistance genes were more abundant in *A. mellifera* from Japan and Switzerland than in *A. mellifera* from China. Thus, the composition of the resistome is mainly different between *A. cerana* and *A. mellifera*, while it is conserved within species across regions.

### Country-wide and location-specific core resistome

**Fig. 3:**
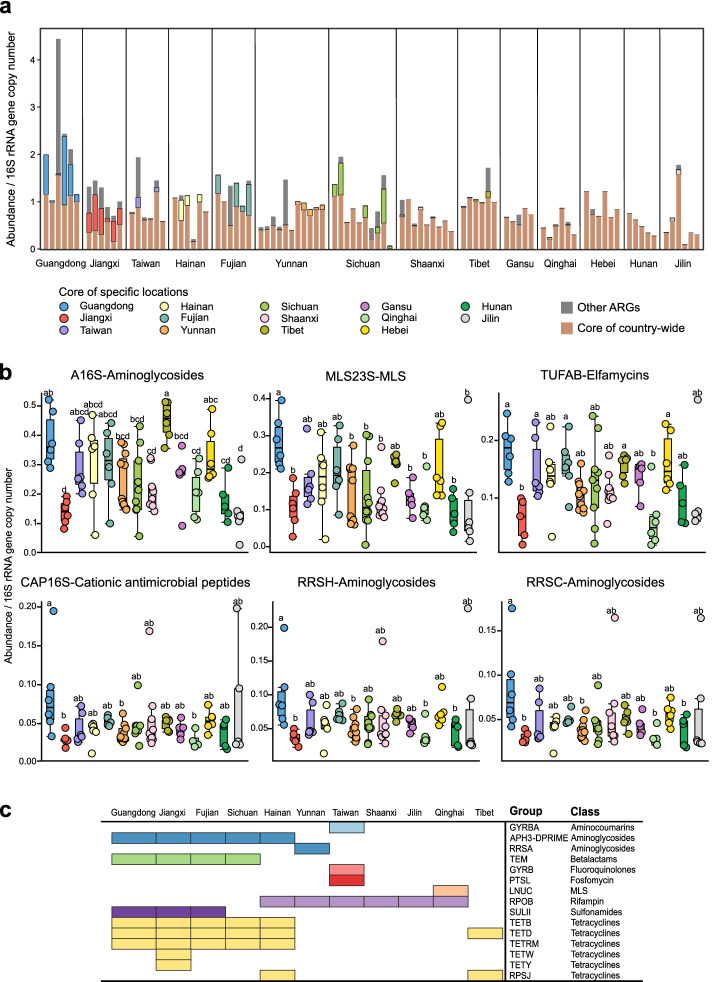
Core and location-specific ARG components of *A. cerana*. **a** Relative abundance of the core ARGs detected in > 50% of *A. cerana* samples (core of country-wide) and those detected in > 50% samples only from certain locations (core of specific locations). Other ARGs occurred only in < 50% samples of any sampling sites. **b** Distribution of the six group of ARGs (Core of country-wide) in bees from different locations. Different letters (a, b, c, d) above each bar stand for statistical differences between *A. cerana* sampling sites (LSD test, *P* < 0.05). **c** The presence of the core ARG groups specific to different locations

Since the gut microbiota of *A. cerana* is relatively consistent across China (Figure S1), we wondered if the ARG components are stable across locations. To characterize the ARGs that were stable among *A. cerana* samples across China, we identified core ARGs detected in > 50% of all samples (country-wide), as well as among sample sets from different regions (region-specific). This followed the definitions from previous resistome studies [34]. We identified six groups of ARGs widespread in > 50% of *A. cerana* samples across all sampling sites. They were defined as “core of country-wide” ARGs, including genes conferring resistance against aminoglycosides (A16S, RRSC, RRSH), cationic antimicrobial peptides (CAP16S), MLS (MLS3S), and elfamycins (TUFAB) (Fig. 3a). Overall, the relative abundance of A16S and MLS23 was higher than that of the other country-wide core groups. All six country-wide ARGs were abundant in *A. cerana* from Guangdong, while all country-wide ARG groups were rare in samples from Jiangxi (Fig. 3b).

In addition, 15 groups of ARGs were specifically present in a high proportion (> 50%) in different locations and were defined as “core of specific locations” ARGs. They consisted of genes conferring resistance against aminoglycoside, beta-lactam, rifampin, sulfonamide, and tetracycline (Fig. 3c, Figure S3). Although *A. cerana* samples from Jiangxi had a low level of country-wide ARGs, specific core ARGs accounted for a large proportion of their resistome (Fig. 3a). Moreover, Jiangxi had the largest number of specific core ARGs (8 groups), which were also prevalent in *A. cerana* from Guangdong, Hainan, and Fujian (Fig. 3c). Interestingly, GYRBA (aminocoumarins), GYRB (fluoroquinolones), and PTSL (fosfomycin) were specific core ARGs in samples from Taiwan.

### Core and transferrable ARGs are carried by different gut bacteria

**Fig. 4:**
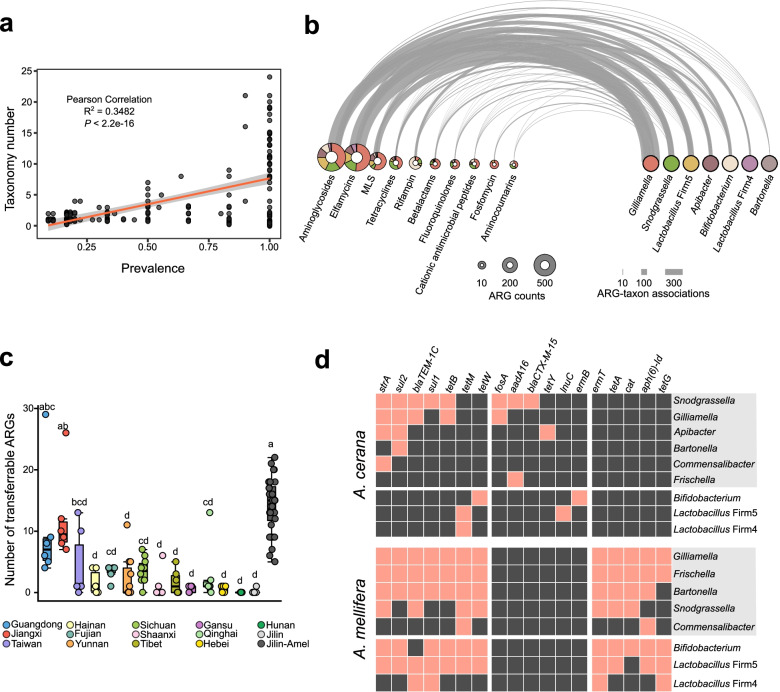
Core and transferrable ARGs are carried by different gut symbionts specific to *A. cerana* and *A. mellifera*. **a** Pearson correlations between the prevalence of each group of core ARGs (both core of country-wide and specific locations) and the number of bacterial species carrying this ARG group in all *A. cerana* samples. The shaded area represents the 95% confidence region. **b** Different classes of ARGs are carried mainly by the core bacterial genera specific to *A. cerana*. Each arc represents the link between a group of ARGs from each class and the bacterial species in each genus. The pie charts show the distribution of bacterial genera in each class of ARGs. The sizes of the pie charts are proportional to the prevalence of each class of ARGs, and the thickness of the lines is proportional to the number of connections (ARG group-bacterial species). **c** Box plots showing the number of transferrable ARGs per honeybee gut sample. Different letters (a, b, c, d) above each bar stand for statistical differences between sampling sites (LSD test, *P* < 0.05). **d** The presence (pink squares) and absence (black squares) transferrable ARGs in different bacterial genus in *A. cerana* and *A. mellifera* gut samples. Gram-negative are marked with grey shades

To explore the contributions of different gut bacteria to the resistome, the distribution of ARGs among gut bacteria was assessed by counting the identified ARG-taxon associations (see “Methods”). We traced the origin of all country-wide and specific core ARGs. We found that the same group of ARGs could be carried by different bacterial species (Dataset S3), and the prevalence of ARG groups positively correlated with the bacterial taxonomy numbers at the species level (Fig. 4a). Most of the ARGs were carried by the core bacterial genera in the gut of *A. cerana*, and the aminoglycoside resistance genes showed the broadest taxonomic ranges and highest frequency (Fig. 4b). Interestingly, ARGs were mainly carried by *Gilliamella*, especially those for resistance to fosfomycin (88%), beta-lactams (73%), fluoroquinolones (63%), and MLS (57%). In contrast, rifampicin resistance genes were contributed mainly by *Bifidobacterium*, and 37% of ARGs against tetracyclines originated from *Snodgrassella*.

Since ARGs were harbored by multiple gut species, we then identified potential transferrable ARGs from assembled metagenomic contigs using ResFinder [28]. A total of 100 ARGs with transfer potential were identified in honeybee gut samples (Dataset S4). Interestingly, Guangdong and Jiangxi provinces with the highest ARG richness also possessed more transferrable ARGs, but they were barely identified in Hunan and Jilin provinces (Fig. 4c). The richness of transferrable ARGs was higher in *A. mellifera* than in *A. cerana*. Seven transferrable ARGs were prevalent in both *A. cerana* and *A. mellifera*, which were shared by all bacterial members in the gut of *A. mellifera* (Fig. 4d). However, in *A. cerana*, *tetM*, and *tetW* were present only in gram-positive *Bifidobacterium* and *Lactobacillus*. Consistently, genes specific to *A. mellifera* were carried by almost all gut bacteria. In *A. cerana*, *Snodgrassella* and *Gilliamella* were the major contributors to transferrable ARGs. Notably, *strA* and *sul2* genes were detected in the *A. cerana*-specific gut member *Apibacter*. The *strA*, *strB*, and *sul2* genes are always organized as an antibiotic resistance gene cluster widely distributed in plasmids and chromosomally integrated elements [35]. We then identified the genetic arrangement of transferrable ARGs and associated mobile gene elements on the assembled contigs in *A. cerana* gut samples.

### IncQ plasmid-mediated *sul2-strA-strB* transmission was frequently identified in *A. cerana*

**Fig. 5:**
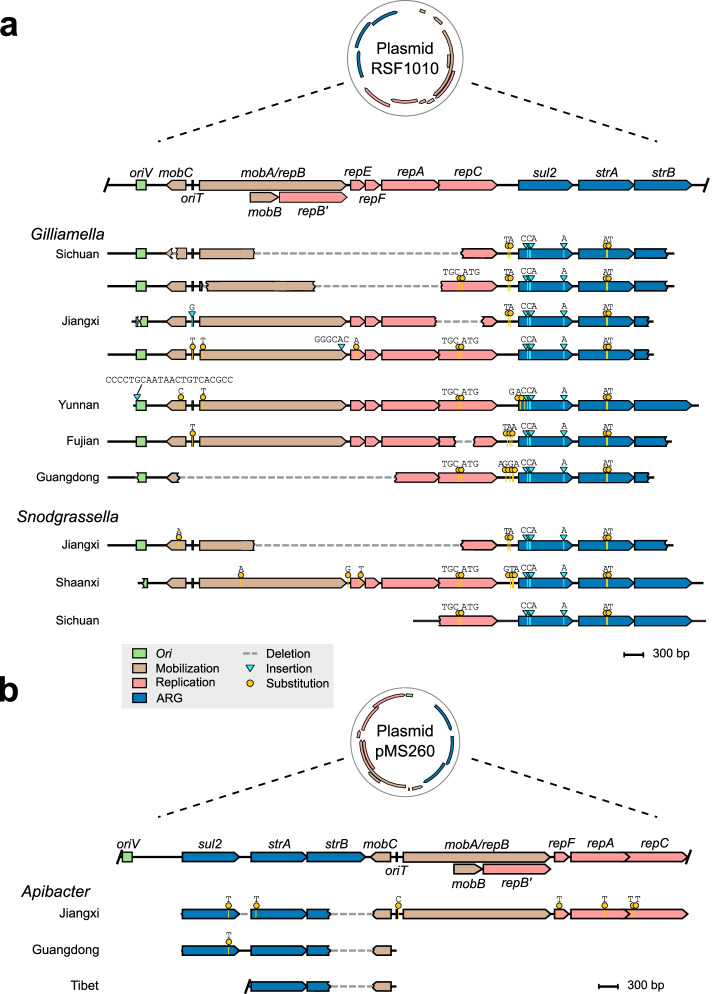
The *sul2*-*strA*-*strB* gene cluster in bee gut bacteria originates from different IncQ-family plasmids. Genetic map of gut bacteria contigs carrying the *sul2*-*strA*-*strB* gene cluster compared with the scaled linear map of the RSF1010 (**a**) and the pMS260 (**b**) plasmids of IncQ-family. Homologous *ori* genes, ARGs, and genes required for mobilization and replication are indicated by different colors. Point mutations and insertions when comparing with the plasmid sequences are indicated on each contig of assembled contigs in samples from different sampling locations. (The position of the mutations and insertions are indicated on each assembled contig from different samples. Dashes represent deletion in the nucleotide sequence)

We found that the *sul2*, *strA*, and *strB* genes also co-occurred in the contigs of the *Gilliamella* and *Snodgrassella* strains. Moreover, the contigs contained genes for the origin of replication (*oriV*), mobilization (*mobABC*), and replication (*repABC*). Interestingly, these genes formed a genomic region highly syntenic to the IncQ plasmid RSF1010, and they were detected in strains even from different geographic locations (Fig. 5a). Although almost all sequences from the symbiont contigs were identical to those of RSF1010, deletions, insertions, and substitutions were detected along the loci. Notably, the *sul2* and *strA* genes were detected in complete forms in all contigs, but the *strB* genes were always truncated. Large fragments of deletions were frequently detected inside the integration regions, mainly spanning the *repBAC* region and the *strB* gene from all contigs, and the deletions inside integration regions were flanked by short homologous sequences.

Furthermore, identical point mutations in *sul2*, *strA*, and even in the intergenic regions were detected in *Gilliamella* and *Snodgrassella* from various locations, suggesting that these mutations occurred before the integrations or were horizontally transferred between different bacterial species. Another gene cluster was found in the *A. cerana*-specific *Apibacter* contigs from Jiangxi, Guangdong, and Tibet, nearly identical to pMS260, a broad-host-range IncQ family plasmid. Similarly, the *strB* and *mobC* genes were incomplete due to partial deletions (Fig. 5b).

### Experimental validation of the IncQ-mediated transmission of ARGs

**Fig. 6:**
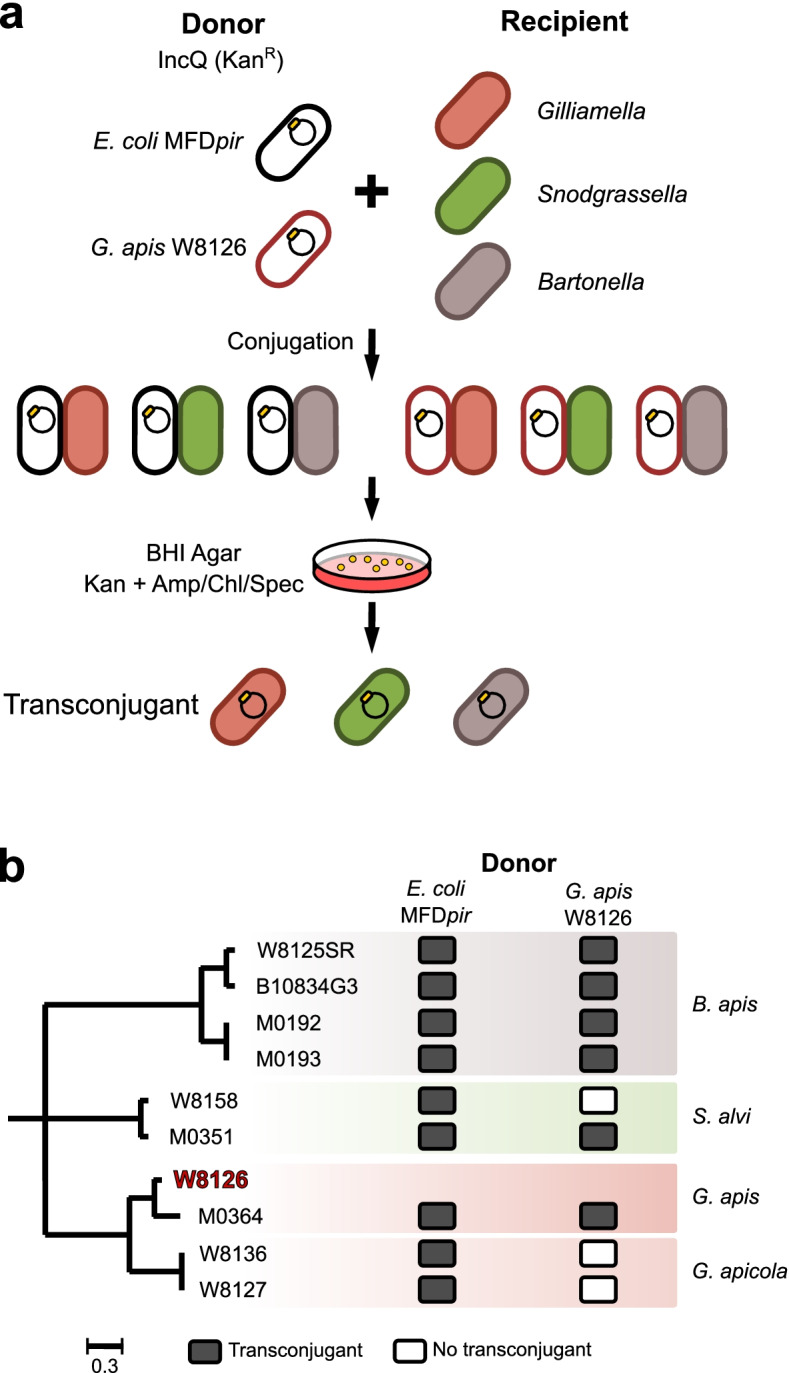
IncQ plasmid transmission between honeybee-specific gut symbionts. **a** Experimental procedure for in vitro conjugation. IncQ plasmid pBTK519 (Kan^R^) was used for transferability test between different donor and recipient strains. Donor strains of *E. coli* MFD*pir* and *G. apis* W8126 harboring the pBTK519 were co-incubated with strains from three honeybee gut bacterial genus, *Gilliamella*, *Snodgrassella* and *Bartonella*, as recipients. BHI agar supplemented with kanamycin and another designated antibiotics were used for transconjugants selection. **b** Experimental results of in vitro conjugation. The phylogenetic tree was constructed by maximum-likelihood method (RAxML) based on the whole genomes of isolated strains. The donor *G. apis* strain W8126 is shown in red

Thus far, our results have shown the widespread transferrable ARGs in *A. cerana* gut symbionts, and the horizontal transfer of ARGs might be associated with the IncQ plasmid as a potential vector. Therefore, we tested whether ARGs could be transferred between bee gut bacteria via the IncQ plasmid. Since IncQ is a non-conjugative but mobilizable plasmid that requires a mating pair channel encoded in bacterial host chromosomes to fulfill its transfer, only donor strains possessing a T4SS can transfer IncQ plasmids between different bacteria. We searched for T4SS component genes in honeybee gut strains, identifying them in a few strains from *Gilliamella*. We then used *G. apis* W8126 as the donor strain to test the transferability of the IncQ plasmid between different gut symbionts. Phylogenetically different strains from *Gilliamella*, *Snodgrassella*, and *Bartonella*, which are gram-negative core members in the bee gut, were used as potential recipients, and *E. coli* MFD*pir* was included as a positive control of the donor strain [36]. First, we tested the natural antibiotic sensitivity of nine recipient strains for the discrimination of transconjugants in subsequent conjugation assays (Dataset S5). Then, we introduced the plasmid pBTK519 that was genetically assembled with the RSF1010 backbone carrying kanamycin resistance into the donors (Fig. 6a). After coculturing the donor and recipient strains for 16 h, we evaluated the conjugation events using selective plates supplemented with different antibiotics. We found that *E. coli* MFD*pir* could deliver the IncQ plasmid to all recipient strains. Although successful conjugative transfer was detected in all *Bartonella* strains, only *S. alvi* M0351 and *G. apis* M0364 could receive the plasmid when using *G. apis* W8126 as the donor (Fig. 5b). No transconjugants were detected in two strains from the *G. apicola* species, suggesting a low conjugation efficiency. Thus, our experiments indicated that the mobilizable IncQ plasmid could be transferred between honeybee gut symbionts, contributing to the broad dissemination of ARGs in different gut bacteria.

## Discussion

The gut microbiome has attracted great attention as it functions as a reservoir and potential ARG source [37]. Although the *A. cerana* gut composition appears to have low variations among different geographical regions (Figure S1), a clear location-dependent resistance pressure was seen in our study. This pressure might be caused by honeybees being subjected to geographically different antibiotic burden from the local environment [12]. During their usual foraging activities, honeybees can cover wide areas where agricultural, industrial, and other anthropogenic activities occur. Therefore, honeybees are likely to be exposed to contaminated environmental sources, such as pollen, nectar and water. There are positive associations between ARG abundance in beehive products and anthropogenic environments, suggesting that ARGs might originate from the honeybee foraging environment [38]. Our results indicated that the gut resistome differs between the two honeybee species and among *A. cerana* from various geographic locations. Previous studies have shown that *A. mellifera* has a larger and more diverse gut community than *A. cerana* [33], which is consistent with our findings (Figure S1). These features may contribute to the observed higher ARG abundance and diversity in *A. mellifera*. In addition, *A. mellifera* carry more tetracycline and sulfonamide resistance genes than *A. cerana* (Fig. 2a), which could be caused by different breeding environment and habits. For example, *A. mellifera* are suitable for breeding in plain areas with concentrated honey sources, and the activity range of *A. mellifera* is closer to human beings [39]. Moreover, it may be contributed by the history of using tetracycline and other related drugs to control bee diseases [40].

The gut microbiome of *A. cerana* is dominated by six country-wide ARGs prevalent in samples across China. Notably, three of these ARGs are aminoglycoside resistance genes. Aminoglycosides are natural antibiotics derived from actinomycetes and are frequently administered to treat bacterial infections [41]. Aminoglycoside resistance genes are detected frequently in rivers [42], livestock [30], and the human gut [43]. Moreover, recent evidence has suggested that other elements (e.g., heavy metals) can select and stimulate the stabilization of aminoglycoside resistance genes [44].

Our results showed that most ARGs within the *A. cerana* core resistome were maintained in the core gut members of honeybees (Fig. 4b). The two proteobacteria, *Gilliamella* and *Snodgrassella*, in the bee gut contributed most to the core resistome. Most ARGs were mainly carried by *Gilliamella*, while *Snodgrassella* was the major carrier of tetracycline resistance genes. A previous screening of the honeybee gut also showed that most of the tetracycline-resistant clones were *Snodgrassella*, which harbored tetracycline resistance loci at high frequencies [10]. However, the distinct resistome profiles of different locations were unlikely to be caused merely by microbiome variance since the gut community composition was discrepant with that of the resistome by Procrustes analysis (Fig. 2g, h). Compared to that of *A. mellifera*, the gut resistome of *A. cerana* consists of fewer ARGs and less ARG diversity. Even after taking the large gut bacterial size and diversity into account, the normalized abundance of ARGs in *A. cerana* was lower. This result suggests that *A. mellifera* was under a higher antibiotic resistance pressure. *A. mellifera* is more intensively managed than *A. cerana*, and there was a long history of oxytetracycline use to control *A. mellifera* larval diseases in the USA [10]. Furthermore, *A. mellifera* is the most frequent crop floral visitor, while *A. cerana* visits local plant species more often [45]. The pollination preference of the two species might also contribute to the varied resistome profile.

Transferable ARGs were the dominant driving force for the overall dissemination of antibiotic resistance. We found that gut samples harboring more ARGs also possessed more transferable ARGs, suggesting that there might be an environmental stress, such as antibiotic selection pressure, that maintains the ARGs [46]. Correspondingly, our results showed that transferrable ARGs were present in all *A. cerana* samples from different locations; however, the load was much lower than that in *A. mellifera* samples. We found syntenic resistance loci with high sequence similarity across bee gut bacteria. These contigs were found in samples even derived from different districts, indicating a high potential for horizontal transfer between bacterial hosts and environments. In particular, two sets of contigs particularly widespread between hosts harbored a *sul2*-*strAB* cluster. Previous studies have detected a high prevalence of streptomycin resistance genes in bees from the USA, and the *strAB* genes are associated with the Tn5393 transposon in *Snodgrassella* [8].

The association of ARGs with mobile elements, such as plasmids and transposons, is critical to facilitate the spread of ARGs between environments [46]. We identified that the nucleotide sequences of the whole genomic region with flanking replication and mobility genes were almost identical to those from the IncQ plasmids (Fig. 5). We found that the genomic regions of *A. cerana* symbionts were essentially identical to two plasmids belonging to the IncQ-1 subgroup. This finding indicates that the IncQ plasmids are integrated into the chromosome of bee gut bacteria, as found in *Salmonella enterica* [47] and *Vibrio cholerae* [48]. Interestingly, we detected sequence deletions in the integrated plasmid fragments, especially in the *strB* and replicon modules. It has been shown that the IncQ plasmid can evolve “satellite plasmids” with replicon deletions, which promises an immediate fitness advantage enabling the maintenance and further transmission of antibiotic resistance traits [49]. In our findings, deletions inside integration regions were flanked by short homologous sequences, as found in the satellite plasmid, suggesting that plasmid evolution might participate in the dissemination of ARGs [49]. In addition, the *sul2-strA-strB* gene cluster was detected in different honeybee gut bacteria, and they were probably derived from different sources of IncQ plasmid origin. We found that the inserted plasmid was widely distributed and persisted almost unchanged across indigenous honeybee symbionts, and this phenomenon was also observed in *Salmonella* from bovine and human sources [35]. Accordingly, our in vitro conjugation assays demonstrated that all tested honeybee gut species were successful recipients of the IncQ plasmid. However, several recipient strains failed in plasmid acquisition despite multiple attempts when *G. apis* W8126 was used as the donor. Specifically, the conjugation efficiency was extremely low for two phylogenetically distant strains (W8136 and W8127), which might be due to genetic divergence [50] or the different restriction modification systems causing genetic isolation [51].

## Conclusions

In this study, we provided a comprehensive overview of the distribution of antibiotic resistance elements in honeybees across China, highlighting the role of the bee microbiome as a reservoir of resistance genes and a potential bioindicator of local antibiotic pressure. Horizontal transfer occurs widely among native gut symbionts, promoting dissemination of antibiotic resistance between honeybee gut bacteria and environmental species. Future works using the honeybee model system could assist the exploration of resistance spread driven by mobile elements in the gut environment and the in vivo evolution of plasmid-mediated antibiotic resistance, which alleviates the fitness costs and favors persistence and propagation.

## Supplementary Information

**Additional file 1: Figure S1**. Gut composition and diversity in *A. cerana* and *A. mellifera* samples. **Figure S2**. Normalized abundance of the ARG classes in *A. cerana* and *A. mellifera* gut samples from different locations. **Figure S3**. Relative abundance of ARGs at the class level in *A. cerana* and *A. mellifera* gut samples from different countries. **Figure S4**. Normalized abundance of location-specific core ARG groups in *A. cerana* gut samples from different locations.

**Additional file 2: Dataset S1**. List of honeybee samples in this study.

**Additional file 3: Dataset S2**. Normalized abundance of ARGs in each metagenomic sample.

**Additional file 4: Dataset S3**. Taxonomic assignment of ARG reads.

**Additional file 5: Dataset S4**. Taxonomic assignment of transferrable ARGs in each sample.

**Additional file 6: Dataset S5**. Detailed information of antibiotic susceptibility of different honeybee gut strains.

## Data Availability

Sequencing data of the metagenomes has been deposited under BioProject PRJNA705951, PRJNA786261, and PRJNA787435.

## Abbreviations

ARGs: Antibiotic resistance genes
CTAB: Cetyl trimethylammonium bromide
T4SS: Type IV secretion system
BHI: Brain heart infusion
DAP: Diaminopimelic acid
MLS: Macrolide-lincosamide-streptogramin
