# Supplementary material for: Geographical resistome profiling in the honeybee microbiome reveals resistance gene transfer conferred by mobilizable plasmids
Source: Microbiome. 2022 May 3;10:69. doi: 10.1186/s40168-022-01268-1 (PMC9063374; doi:10.1186/s40168-022-01268-1)
Supplement: Supplementary file 2 — Additional file 1: Figure S1. Gut composition and diversity in A. cerana and A. mellifera samples. Figure S2. Normalized abundance of the ARG classes in A. cerana and A. mellifera gut samples from different locations. Figure S3. Relative abundance of ARGs at the class level in A. cerana and A. mellifera gut samples from different countries. Figure S4. Normalized abundance of location-specific core ARG groups in A. cerana gut samples from different locations. [file 40168_2022_1268_MOESM1_ESM.pdf]

Supplementary Information

Geographical resistome profiling in honeybee microbiome reveals resistance gene transfer conferred by mobilizable plasmids

Huihui Sun, Xiaohuan Mu, Kexun Zhang, Haoyu Lang, Qinzhi Su, Xingan Li, Xin Zhou, Xue Zhang*, Hao Zheng*

Fig. S1. Gut composition and diversity in *A. cerana* and *A. mellifera* samples.

Fig. S2. Normalized abundance of the ARG classes in *A. cerana* and *A. mellifera* gut samples from different locations.

Fig. S3. Relative abundance of ARGs at the class level in *A. cerana* and *A. mellifera* gut samples from different countries.

Fig. S4. Normalized abundance of location-specific core ARG groups in *A. cerana* gut samples from different locations.

Dataset. S1. List of honeybee samples in this study.

Dataset. S2. Normalized abundance of ARGs in each metagenomic sample.

Dataset. S3. Taxonomic assignment of ARG reads.

Dataset. S4. Taxonomic assignment of transferrable ARGs in each sample.

Dataset. S5. Detailed information of antibiotic susceptibility of different honeybee gut strains.

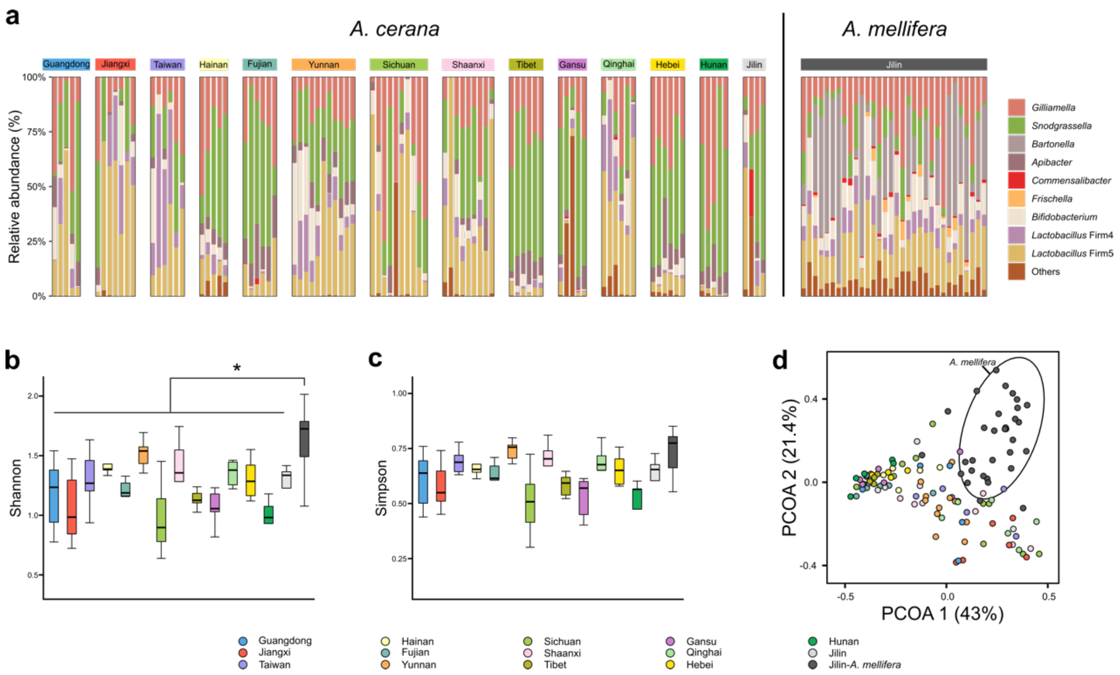

Fig. S1. Gut composition and diversity in *A. cerana* and *A. mellifera* samples. (a) Stacked bar charts showing the relative abundance of different genera of gut bacteria in each bee individual. (b-c) Box plots showing the shannon (b) and simpson (c) diversity of the gut community in each sample. (d) PCoA based on the Bray-Curtis distances of microbiome compositions.

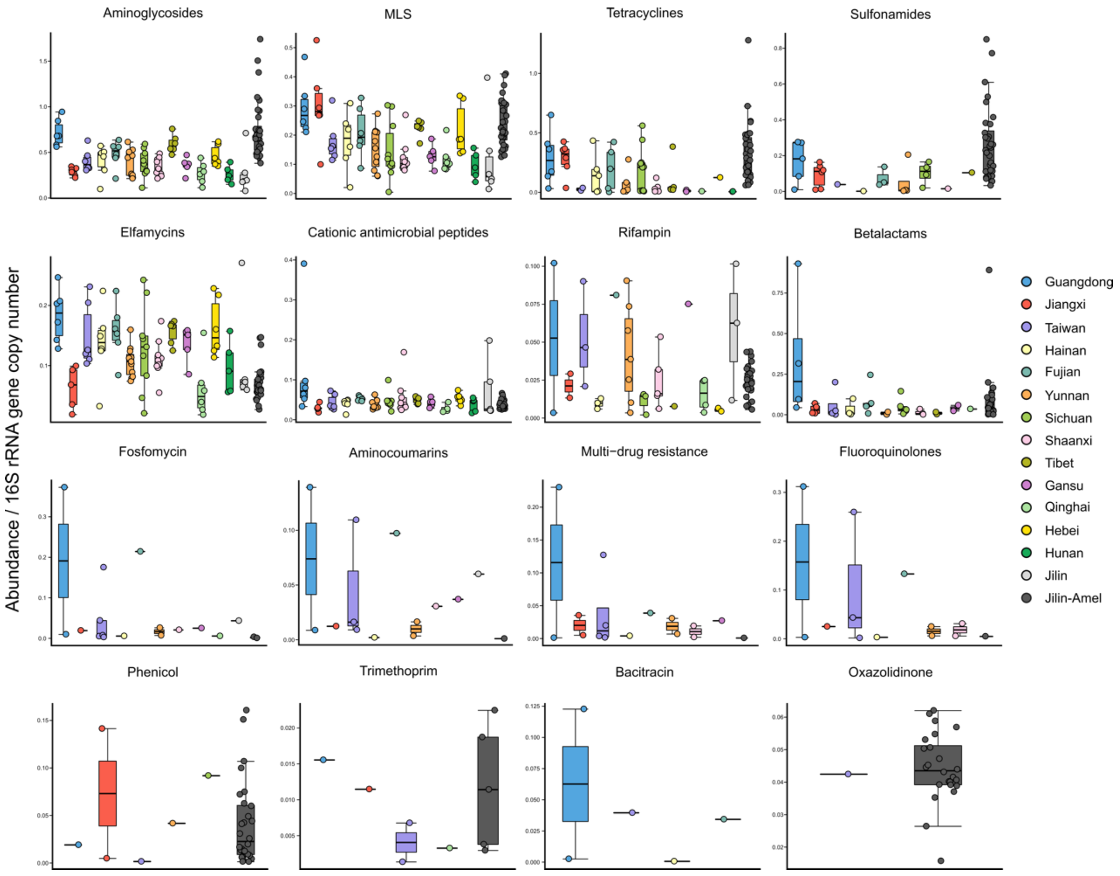

Fig. S2. Normalized abundance of the ARG classes in *A. cerana* and *A. mellifera* gut samples from different locations.

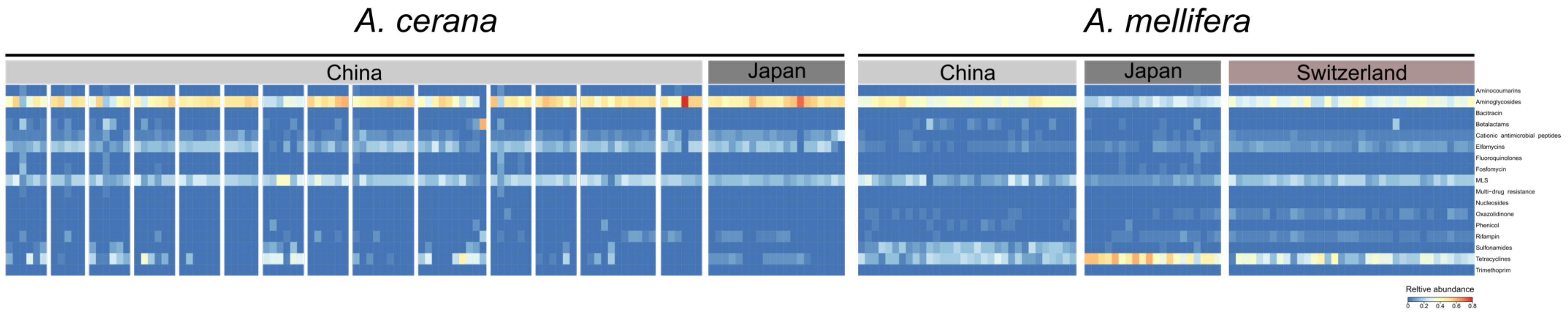

Fig. S3. Relative abundance of different classes of ARGs in each *A. cerana* and *A. mellifera* gut samples from different countries.

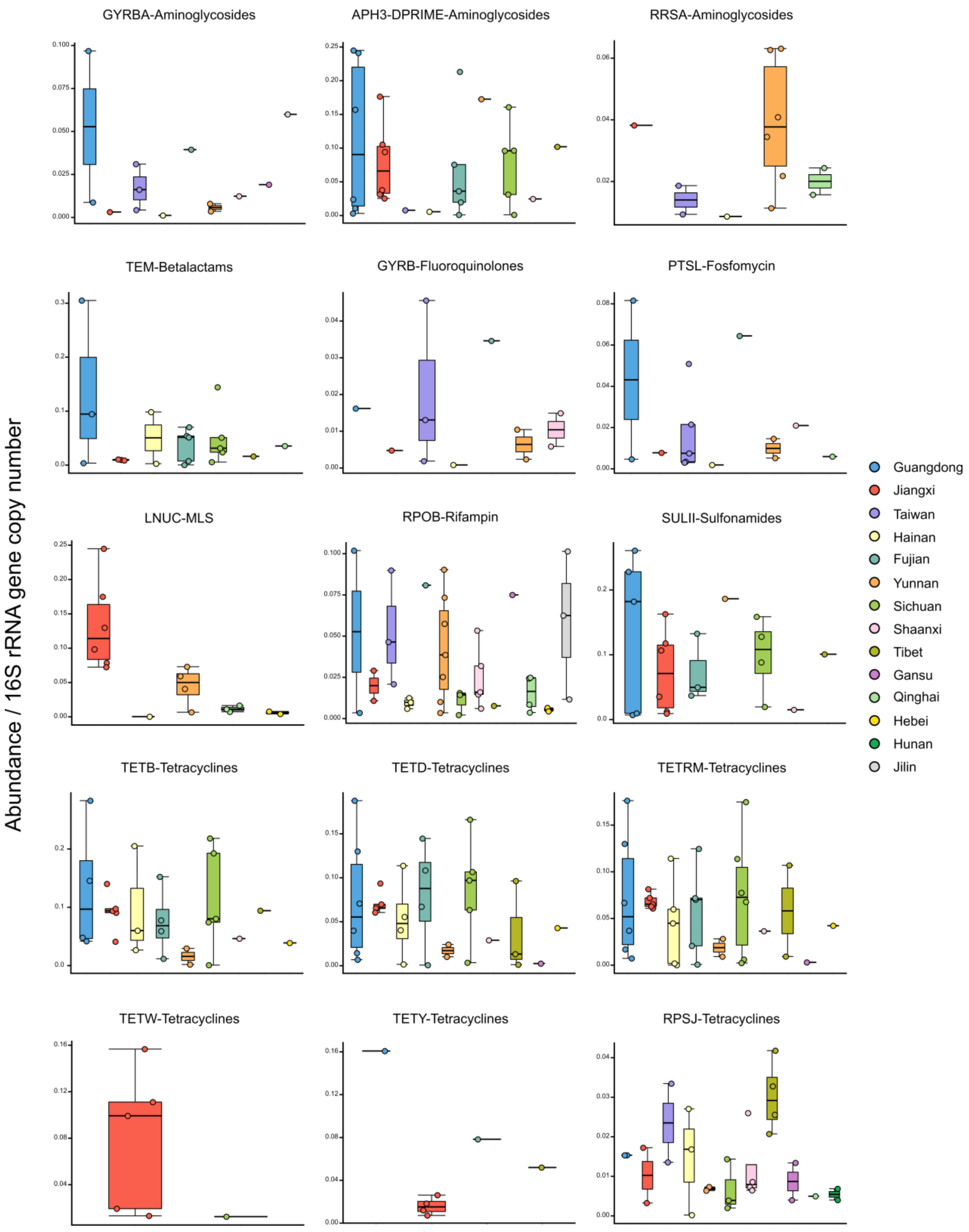

Fig. S4. Normalized abundance of location-specific core ARG groups in *A. cerana* gut samples from different locations.
